# SARS-CoV-2 test positivity rate in Reno, Nevada: association with PM2.5 during the 2020 wildfire smoke events in the western United States

**DOI:** 10.1038/s41370-021-00366-w

**Published:** 2021-07-13

**Authors:** Daniel Kiser, Gai Elhanan, William J. Metcalf, Brendan Schnieder, Joseph J. Grzymski

**Affiliations:** 1 Center for Genomic Medicine, Desert Research Institute, Reno, NV USA; 2 Washoe County Health District Air Quality Management Division, Reno, NV USA; 3 Renown Health, Reno, NV USA

## Abstract

**Background:**

Air pollution has been linked to increased susceptibility to SARS-CoV-2. Thus, it has been suggested that wildfire smoke events may exacerbate the COVID-19 pandemic.

**Objectives:**

Our goal was to examine whether wildfire smoke from the 2020 wildfires in the western United States was associated with an increased rate of SARS-CoV-2 infections in Reno, Nevada.

**Methods:**

We conducted a time-series analysis using generalized additive models to examine the relationship between the SARS-CoV-2 test positivity rate at a large regional hospital in Reno and ambient PM2.5 from 15 May to 20 Oct 2020.

**Results:**

We found that a 10 µg/m^3^ increase in the 7-day average PM2.5 concentration was associated with a 6.3% relative increase in the SARS-CoV-2 test positivity rate, with a 95% confidence interval (CI) of 2.5 to 10.3%. This corresponded to an estimated 17.7% (CI: 14.4–20.1%) increase in the number of cases during the time period most affected by wildfire smoke, from 16 Aug to 10 Oct.

**Significance:**

Wildfire smoke may have greatly increased the number of COVID-19 cases in Reno. Thus, our results substantiate the role of air pollution in exacerbating the pandemic and can help guide the development of public preparedness policies in areas affected by wildfire smoke, as wildfires are likely to coincide with the COVID-19 pandemic in 2021.

## Introduction

During the second half of the summer of 2020, two crises converged on residents of the western United States: the second wave of the COVID-19 pandemic and widespread wildfires. As a result of the wildfires, many residents had prolonged exposure to smoke containing elevated levels of particulate matter 2.5 µm in diameter or smaller (PM2.5). It has been suggested that even a moderate magnitude wildfire smoke event may increase the impact (incidence or mortality) of COVID-19 by ~10% (ref. [1]), but this has not yet been adequately verified.

Air pollution is detrimental to health in general and to respiratory health in particular (ref. [2, 3]). While air pollution is composed of various gases and particles of different sizes, PM2.5 is likely the chief mediator of its ill effects (ref. [4, 5]). PM2.5 increases susceptibility to respiratory viruses via modified immune responses, including systemic and airway inflammation (ref. [6, 7]). Moreover, it has been shown that small particulates can enhance the spread and survival of bacterial, fungal, and viral bioaerosols (ref. [8, 9]), including bioaerosols containing SARS-CoV-2 (ref. [10]). An association between particulate matter (PM) and daily mortality from SARS-CoV-1 was reported in 2005 (ref. [11]). Copat et al. provide a systematic review of the role of air pollution and its effect on the spread and lethality of COVID-19 (ref. [12]).

**Fig. 1:**
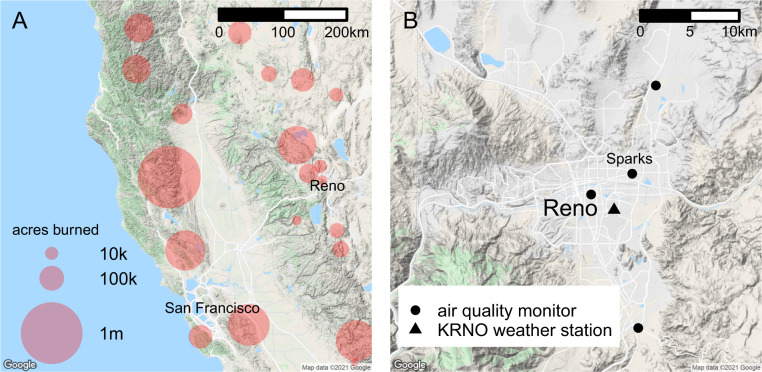
Maps of wildfire and air quality monitor locations. **A** Map of major wildfires occurring in the region during our study period, 15 May to 20 Oct 2020, that likely impacted air quality in Reno, Nevada. The size of the points indicates the number of acres burned in each wildfire. Additional information on wildfire names, locations, start and end dates, and size measured in acres can be found in Table S1. **B** Map of air quality monitors in Reno. The location of the KRNO weather station is also shown. API calls for Google Maps provided in refs. [38, 39].

PM2.5 specifically from wildfires has been shown to be detrimental to respiratory health as well (ref. [13–15]). Washoe County, in Northern Nevada (NV), was heavily exposed to smoke from the 2020 wildfires, while simultaneously experiencing increasing numbers of COVID-19 cases. See Table S1 for a list of major wildfires that affected air quality in Washoe County during our study period, and see Fig. 1A for a corresponding map. The county has a population of 480,000, with at least 75% of residents living in the Reno-Sparks metropolitan area (Reno). Reno’s location in an intermountain valley restricts the dispersion of pollutants (ref. [16]), possibly increasing the magnitude of exposure. The largest healthcare system in Washoe County is Renown Health (Renown), whose laboratory-confirmed about 25% of the county’s COVID-19 cases.

Our objective was to determine whether wildfire PM2.5 is associated with an increased rate of SARS-CoV-2 infections. Thus, we examined the association between positive COVID-19 cases at Renown and ambient concentrations of PM2.5 in Reno, NV during a time period that included a severe, prolonged smoke event caused by the 2020 wildfires.

## Methods

### Data

Preliminary PM2.5 concentrations were obtained via the Environmental Protection Agency’s (EPA’s) internet database (www.epa.gov/airdata) from four air quality monitors located in Reno and Sparks (see Fig. 1B for exact locations). These federal equivalent method (FEM) PM2.5 monitors were hourly Beta Attenuation Monitors (Met One BAM 1020s) with a very sharp cut cyclone (VSCC). To estimate the daily exposure of the population to PM2.5, we used a weighted average of the daily concentrations reported by each monitor, with the weights proportional to the count of Renown patients living within five kilometers of each monitor. If measurements of PM2.5 were missing at a particular monitor, we used a weighted average of the remaining monitors. PM2.5 concentrations were missing for all monitors on 29 Sep 2020 due to a change in AirNow’s file transfer protocol (FTP) address that occurred that afternoon, causing data not to be reported. We obtained the missing values directly from the Washoe County air quality management division. Six negative values for PM2.5 concentration were replaced with a value of zero before obtaining the weighted average.

Temperature and humidity data were obtained from the KRNO weather station (via mesowest.utah.edu), which is located near the Reno airport (Fig. 1B), and records data every five minutes. These values were averaged by date in order to obtain daily means. However, some 5 minute measurements were missing. These missing values were ignored unless they represented more than 25% of the values for a given day. This occurred on only 1 day, 9 Aug 2020. For this day, mean temperature and mean humidity was estimated as the average of the daily means from the day prior and the day after.

SARS-CoV-2 nucleic acid amplification (NAA) test results and patient demographic data were obtained from Renown. During the study period, no significant shortages of SARS-CoV-2 tests were observed and testing indications followed published CDC and Washoe County Health District clinical guidelines. For our analysis, we included all available types of SARS-CoV-2 NAA tests except one. We excluded this test because it was used only briefly during our study period, it was administered in very large numbers during that brief period, it had a very low positivity rate, and it was rarely utilized during the period most heavily affected by wildfire smoke (16 Aug to 10 Oct 2020). For calculating the daily count of positive SARS-CoV-2 NAA test results, we included only a patient’s initial positive test. For calculating the daily total of tests administered, we included the first positive test for patients who tested positive and only one negative test per patient per day of patients who never tested positive from the start of the pandemic (2 Mar 2020) to the end of our available data (21 Oct 2020).

We selected 15 May 2020 as the beginning of our study period, as that appears to be when the number of tests administered stabilized, and our study period ended on 20 Oct 2020.

### Modeling

We used a generalized additive model from the Negative Binomial distribution, rather than from the Poisson distribution, to account for any over-dispersion present in the counts of positive COVID-19 cases. Our base model specification was:

$$log\left( {Y_i} \right) = \beta _0 + \beta _1temp_i + \beta _2Y_{i - 1} + \delta DOW_i + s\left( {time_i} \right) + \log \left( {total_i} \right) + \epsilon _i$$

where *Y*_*i*_ is the count of positive COVID-19 cases on the day *i*, *temp*_*i*_ are the 7-day average of mean temperature, *Y*_*i–1*_ is the count of positive cases on the previous day, *DOW*_*i*_ is an indicator for the day of the week, *s*(*time*_*i*_) is a cubic regression spline of time (measured in the number of days since the start of the study period), and *total*_*i*_ is the total number of tests administered. *β* and *δ* indicate coefficients to be estimated, with *δ* indicating the coefficient specific to a particular day of the week. *∈*_*i*_ is the random error. In this paper, we define the 7-day average for day *i* as the mean of the variable value on days *i* through *i*–6. Similarly, we define the 3-day average for day *i* as the mean of the variable value on days *i* through *i*–2.

Because we used the total number of tests administered as an offset, we essentially modeled the positivity rate, rather than counts of positive cases. The positivity rate has the advantage of being less dependent on the number of SARS-CoV-2 NAA tests administered than the number of positive cases, though it is not completely independent, as changes in the number of tests administered may result in changes in the population being tested.

We adjusted for the temperature to account for any seasonal changes in the SARS-CoV-2 infection rate. The 7-day average of mean temperature was used instead of the daily mean temperature to account for a probable delay between changes in temperature and changes in the infection rate.

**Fig. 2:**
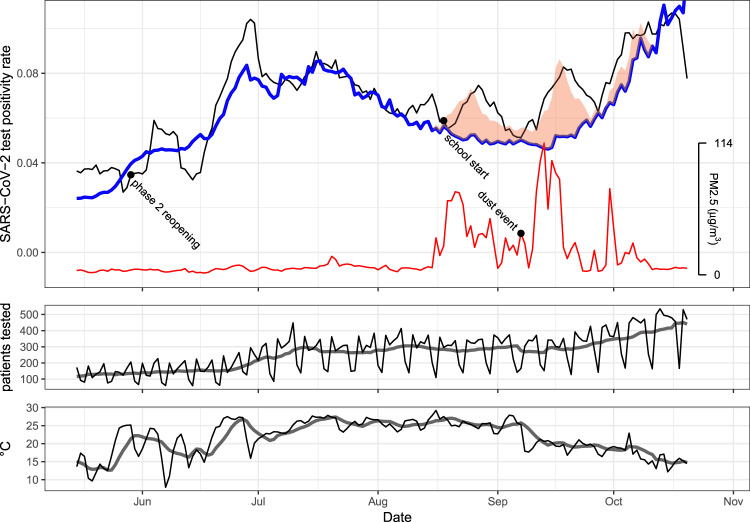
SARS-CoV-2 tests, PM2.5, and temperature over time. Top panel: SARS-CoV-2 test positivity rate from 15 May 2020 to 20 Oct 2020. Expected positivity rates were estimated by the model which included the 7-day average of daily mean PM2.5 as a predictor. The black line indicates the 7-day moving average of the positivity rate (each day averaged with the 3 days prior and the 3 days following). The blue line indicates the expected positivity rate if the concentration of 7-day average PM2.5 during the period 16 Aug 2020 to 10 Oct 2020 had remained at the average level of the same period in 2019 (4.5 µg/m^3^). The red-shaded region indicates the expected positivity rate based on the actual PM2.5, which is indicated by the red line. For this figure, weekday effects were removed from the model estimates for clarity. Middle panel: Daily number of patients with SARS-CoV-2 NAA tests at Renown is indicated by the thin line, while the 7-day average is indicated by the bold line. Bottom panel: daily mean temperature (°C) is indicated by the thin line, while the 7-day average is indicated by the bold line.

The smooth of time was necessary to control for confounding factors that contribute to changes in the positivity rate, but for which no reasonable data source exists. These confounding factors include the prevalence of the virus in the community at any given time, changes in human behavior, and changes in which patients are being tested. While some information exists on pertinent events that may affect human behavior (such as the Phase 2 reopening of Nevada or the start of school, indicated in Fig. 2), it is uncertain how to specifically model these events. Using a smooth function of time as a predictor allowed us to model the general course of the pandemic, while still examining whether PM2.5 explained particular features of the data.

The autoregressive term *Y*_*i*–1_ was added as a predictor because autocorrelation function (ACF) and partial autocorrelation function (PACF) plots revealed significant autocorrelation at lag 1 of the residuals. This was confirmed by the Durbin–Watson test (*p* = 6.3e−14). After adding this term, the ACF plot, PACF plot, and Durbin–Watson test (*p* = 0.78) indicated that the autocorrelation had been removed.

However, we noticed that when PM2.5 terms were added to the model, we tended to see negative autocorrelation in the residuals at various lags. We concluded that the smooth of time was somewhat overfitting. The software we used automatically determines the degrees of freedom for the smooth term based on restricted maximum likelihood, with an upper limit selected by the user. The default maximum value for degrees of freedom is nine. When we lowered this value to four, the negative autocorrelation disappeared. Thus, we used four as the degrees of freedom for the smooth of time.

We initially investigated the relationship between the positivity rate and PM2.5 using lags 0, 7, and 14 of both daily mean PM2.5 and the 7-day average of daily mean PM2.5. To further refine our understanding of the chronological relationship between PM2.5 and positive SARS-CoV-2 tests, we subsequently tested the association of the remaining lags of daily mean PM2.5 between 0 and 14, as well as lags 0, 3, 6, 9, and 12 of the 3-day averages of daily mean PM2.5.

We then used the method described by Schwartz to fit distributed lag models (DLMs), which constrain the lag effects to follow a polynomial function (ref. [17]). We concluded based on the single-day and three-day average models that the lag effects were likely to follow a quadratic or cubic function. Modifying the number of lags included in the DLM can have a sizable influence on the estimates for each lag, and it is not necessarily clear how many lags should be included. Based on the results of previous models, we assumed that a reasonable quadratic or cubic function for the lags would include (1) significant positive associations at some lags, (2) no large negative associations at large lags, and (3) a near-zero effect at the largest lag.

### Calculating excess cases

We used the model which included the 7-day average of PM2.5 as a predictor to estimate how many fewer COVID-19 cases were likely to have occurred if there had been no wildfire smoke between 16 Aug and 10 Oct 2020. This model was chosen because the lags were selected a priori and it was more parsimonious than the distributed lag models. Below is a description of how we estimated excess cases and found a confidence interval for that estimate.

For our model, the fitted value for a day *i* is:

$$z_i = \hat \beta _0 + \hat \beta _1temp_i + \hat \beta _2Y_{i - 1} + \hat \delta DOW_i + \hat s\left( {time_i} \right) + \hat \beta _3PM2.5_i$$

where *z*_*i*_ is the fitted value on the link scale for day *i*, *PM*2.5_*i*_ is the 7-day average of PM2.5, and the other variables are defined the same as they were previously. Each *z*_*i*_ is necessarily a normally distributed variable, given that the model diagnostics indicate normally distributed residuals (Fig. S1).

We assumed that if there had not been a wildfire smoke event from 16 Aug to 10 Oct 2020, the 7-day average of PM2.5 would have remained at the average level of the same period in 2019, when no major wildfire smoke events occurred. We generated fitted values for each day from 16 Aug to 10 Oct 2020, based on the altered data. We then calculated excess cases as follows:

$$W = \mathop {\sum }\limits_{i = 1}^n Y_i - (exp(z_i) \times total_i)$$

where *W* is the expected number of excess cases for the entire time period, and *Y*_*i*_ is the actual number of cases on day *i*. Day 1 is the first day of the time period of interest (in our case, 16 Aug 2020) and day *n* is the last day (10 Oct 2020). *z*_*i*_ was exponentiated and multiplied by the offset (*total*_*i*_) in order to transform it to the same scale as the response (*Y*_*i*_).

Because of the exponentiation of *z*_*i*_, finding the variance (and hence a confidence interval) for *W* is not straightforward. We thus adopted a resampling approach that uses the distribution of the individual *z*_*i*_:

1. For each day *i*, we selected a random value from the distribution of *z*_*i*_, assuming normality and using the standard errors for each *z*_*i*_ reported by the software. We denote this value as $z_i^ \ast$.
2. We then calculated: $W^ \ast = \mathop {\sum}\nolimits_{i = 1}^n {Y_i - \left( {exp\left( {z_i^ \ast } \right) \times total_i} \right)}$, where W^*^ is a new estimate of excess cases.

Steps 1 and 2 were repeated 10,000 times, and the 2.5th and 97.5th percentiles of the empirical distribution of the W^*^’s were assigned as the lower and upper confidence interval limits of *W*.

### Additional methods

To test the robustness of our results, we made several modifications to the base model and evaluated their separate effects on the results. We (1) increased the maximum degrees of freedom for the smooth of time from four degrees of freedom to nine degrees of freedom, (2) added the 7-day average of relative humidity as a predictor, and (3) replaced the 7-day average of temperature with a 3-day average of temperature.

We used R version 3.6.0 and the R packages mgcv 1.8–28 (ref. [18]) and mgcv.helper 0.1.8 (ref. [19]) for all of our statistical modeling. ggmap 3.0.0 was used to generate maps (ref. [20]). Work on this study was Institutional Review Board exempt by the University of Nevada, Reno Institutional Review Board (#1106618-25) as part of a larger body of work “Interoperability, operational efficiency and quality of care improvements through health data analysis.” The funders had no role in study design, data analysis, decision to publish, or preparation of the manuscript.

## Results

During the study period (159 days), 35,955 individuals were tested at Renown for SARS-CoV-2, of which 2881 (8.0%) tested positive (Table 1). 72% of patients were 30 years of age or older, but the rate of patients testing positive was highest among patients aged 18–29, with a positivity rate of 11.3%. The number of NAA tests administered at Renown increased steadily during our study period, from an average of 130 patients tested per day in the second half of May to 404 patients tested per day in the first half of October (Fig. 2).

**Table 1:** Demographics of the patient population tested for SARS-CoV-2 at renown from 15 May to 20 Oct 2020.

	Patients tested			Percent Positive
	Total	Negative^a^	Positive^b^	
Patients, *N* (%)	35955 (100)	33074 (100)	2881 (100)	8.0%
Male gender, *N* (%)	15798 (44)	14432 (44)	1366 (47)	8.6%
Age^c^, mean (SD)	45.4 (23.0)	45.6 (23.2)	42.3 (20.4)	NA
Age^c^ category, *N* (%)				
<18	4632 (13)	4352 (13)	280 (10)	6.0%
18–29	5598 (16)	4964 (15)	634 (22)	11.3%
30–49	9653 (27)	8729 (26)	924 (32)	9.6%
50–69	10071 (28)	9325 (28)	746 (26)	7.4%
≥70	6001 (17)	5704 (17)	297 (10)	4.9%
Race, *N* (%)				
White	29439 (82)	27483 (83)	1956 (68)	6.6%
Non-white	2881 (8)	2600 (8)	281 (10)	9.8%
Unknown	3635 (10)	2991 (9)	644 (22)	17.7%

^a^Patient had only negative tests results.

^b^Patient had at least one positive test result.

^c^Age in years at the first test, regardless of whether the test was positive or negative.

Air quality was affected by wildfire smoke for 59 days during our study period, with 50 of those days occurring between 16 Aug and 10 Oct 2020 (Fig. 2). Daily mean concentrations of PM2.5 averaged across all four monitors ranged between 1.5 µg/m^3^ (17 Jun 2020) to 114.3 µg/m^3^ (13 Sep 2020). We note that a dust storm event contributed to PM2.5 on 7–8 Sep 2020.

**Fig. 3:**
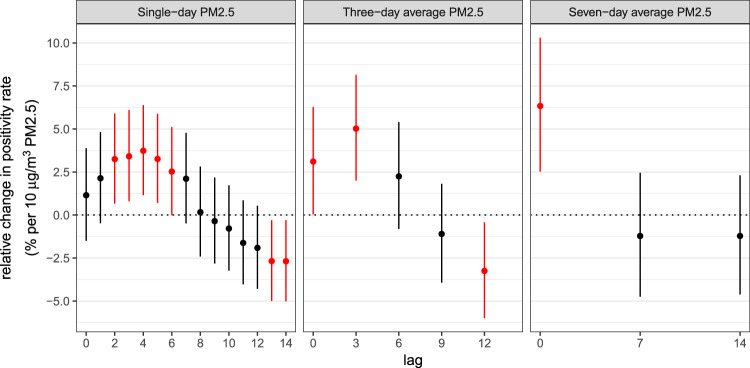
The relative percent change in the SARS-CoV-2 test positivity rate associated with an increase of 10 μg/m^3^ PM2.5 at lags 0–14 of single-day PM2.5 (left panel); lags 0, 3, 6, 9, and 12 of the three-day average PM2.5 (middle panel); and lags 0, 7, and 14 of the 7-day average PM2.5 (right panel). Error bars indicate 95% confidence intervals, which were not corrected for multiple testing.

Using lags selected a priori, we found that a 10 µg/m^3^ increase in the 7-day average of PM2.5 was associated with a 6.3% relative increase in the positivity rate, with a 95% confidence interval (CI) of 2.5–10.3% (Fig. 3). Lag 14 single-day PM2.5 had a significant negative association with the positivity rate. This appears to be due to the limited duration of especially high concentrations of PM2.5 in our data, which causes lag 14 PM2.5 to predict the troughs between the surges in the positivity rate (Fig. S2). After examining other lags of PM2.5, we found positive associations between the SARS-CoV-2 test positivity rate and lags 2–6 of single-day PM2.5, as well as lags 0 and 3 of 3-day average PM2.5 (Fig. 3). Additional negative associations were found for lag 13 of the single-day PM2.5 and for lag 12 of the 3-day average PM2.5.

**Fig. 4:**
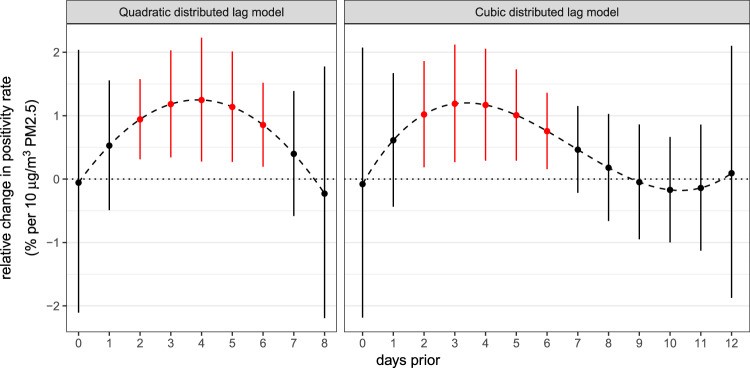
The relative percent change in the SARS-CoV-2 positivity rate associated with an increase of 10 μg/m^3^ in daily mean PM2.5 at each lag, where separate lag effects are considered within the same model and the effects are constrained to follow a quadratic function over lags 0–8 (left panel) and a cubic function over lags 0–12 (right panel). Error bars indicate 95% confidence intervals, which were not corrected for multiple testing.

Based on the results of the single-day and the 3-day average models, we determined that intermediate-length lags had the largest association with the positivity rate. Using the criteria described previously, we selected lags 0–8 for the quadratic DLM and lags 0–12 for the cubic DLM. These models indicated that lags 2–6 have a significant positive association with the positivity rate, with a 10 µg/m^3^ increase in PM2.5 being associated with an ~1% relative increase in the positivity rate on any given lag (Fig. 4).

Based on the model using 7-day average PM2.5 as a predictor, exposure to wildfire PM2.5 accounted for an additional 178 (CI: 149–98) positive COVID-19 cases at Renown alone between 16 Aug and 10 Oct 2020, or an increase of 17.7% (CI: 14.4–20.1%). Fig. 2 compares the expected positivity rate in the presence and absence of wildfire smoke during that time period.

The results of the models in our main analysis are shown in tabular form in Table S2, while the results of the models in our sensitivity analyses are shown in Tables S3–S5, for comparison. A comparison of excess cases estimated in each analysis can be viewed in Table S6. None of the results of the sensitivity analyses meaningfully differed from the results of the main analysis.

## Discussion

We found a large increase in the SARS-CoV-2 test positivity rate at Renown during periods of elevated PM2.5 from wildfires. These results, although based on observational data with their inherent limitations, lend credence to earlier predictions that wildfire smoke would exacerbate the COVID-19 pandemic (ref. [1, 21]). Our findings also bolster arguments that PM2.5 from other sources, such as vehicle traffic or industry, increases susceptibility to SARS-CoV-2. An association of elevated levels of air pollution with increased infectivity and severity of COVID-19 cases was found in populated areas in Italy (ref. [22, 23]), the United States (ref. [24, 25]), England (ref. [26]), China (ref. [27]), and other nations (ref. [23, 28]), but not in Spain (ref. [23, 29]).

A previous study by Meo et al. examining the relationship between PM2.5 and daily new COVID-19 cases in San Francisco, California during the 2020 wildfires found an association of similar direction and magnitude as some of the effects observed in our study—a 5.1% increase in daily cases per 10 µg/m^3^ increase in PM2.5 (ref. [30]). Another study by Leifer et al. observed increased numbers of COVID-19 cases following wildfire smoke events in Orange County, California (ref. [31]). However, our study significantly improved on these studies by controlling for additional covariates: the general prevalence of the virus, which increased over time (not included in Meo et al.); and temperature and the number of tests administered (not included in Meo et al. or Leifer et al.). Thus, we believe that our study greatly strengthens the evidence that wildfire smoke can enhance the spread of SARS-CoV-2.

In addition to the mechanisms mentioned previously, where PM2.5 enhances the pathogenicity of viruses by modifying immune responses and facilitating the transport of the virus into the lungs, a third possible mechanism specific to SARS-CoV-2 may involve the ACE2 receptor, the molecular target for the virus. Elevated concentrations of ambient nitrogen dioxide (NO2) and PM2.5 result in over-expression of the ACE2 receptor in respiratory epithelial cells, possibly increasing the pathogenicity of the virus (ref. [32]). It is unclear whether this mechanism requires long- or short-term exposure to air pollution, or whether in vivo effects might differ from in vitro effects (ref. [33]). However, in vitro studies suggest that relatively short exposure to PM2.5 may induce cellular changes and inflammation (ref. [33, 34]).

Our data did not support a same-day association between the SARS-CoV-2 test positivity rate and PM2.5, and further investigation revealed that the positivity rate was most strongly associated with PM2.5 concentrations two to 6 days prior. Thus, our results are consistent with a relatively short-term, cumulative effect of wildfire PM2.5 exposure on the incidence of COVID-19, possibly mediated by cellular changes and increased infectivity due to PM particles acting as vectors of spread. Other non-biological factors may also play a role: use of air quality monitoring applications is pervasive and encourages people to stay indoors during bad air events, which could enhance the spread of SARS-CoV-2 in indoor public places like restaurants and schools. Conversely, they could also limit the spread of SARS-CoV-2 if public places are mandated to be closed and people are forced to take shelter from the smoke in their own homes. Thus, it is possible that the influence of wildfire smoke on the spread of SARS-CoV-2 depends strongly on human behavior and public policy decisions, like the decision to resume in-person classes. The Washoe County School District implemented a hybrid system of in-person and virtual classes for middle schoolers and high schoolers (students aged 11–18 years), where students attended in-person classes every other day, while elementary schoolers (students aged 5–11 years) continued attending in-person classes every day. However, it is not likely that the reopening of school is an important confounder in our study since the increases in the positivity rate attributed to PM2.5 were temporary surges, rather than a sustained increase as might be expected when students and teachers are perpetually in contact.

Although this study only evaluated positivity rates, it is reasonable to assume that the excess cases due to wildfire PM2.5 resulted in excess mortality. Wildfire smoke has previously been associated with increased all-cause mortality (ref. [35, 36]), and an association between this season’s smoke episode and mortality was observed in Washington state (ref. [37]). However, it is beyond the scope of this study to evaluate the number of COVID-19 deaths attributable to PM2.5.

This study also limited itself to examining exposure to PM2.5, even though wildfire smoke consists of many other potentially harmful compounds, such as coarse PM, carbon monoxide, volatile organic compounds, ozone, and nitrogen oxides. It is possible that some of the associations we observed between PM2.5 and the SARS-CoV-2 test positivity rate could be attributed to these other compounds. Although such confounding would be largely irrelevant from a public policy standpoint, it may have implications for understanding the mechanism behind the increased rate of infections.

In addition, our study has important limitations with regard to our ability to quantify exposure to PM2.5. While four air quality monitors are quite sufficient for estimating outdoor air quality, we were unable to account for variations in individual exposure that may be due to occupation, recreation, or income. Thus, using our method of estimating exposure could result in different estimates of the effect between our study population and other populations who have different occupations, recreational habits, or social statuses.

Reno likely presents a better opportunity to study the association of wildfire smoke with COVID-19 incidence than some of the localities that were directly threatened by wildfires, since Reno was not subject to evacuations that would have prevented exposure to smoke. Reno was also exposed to higher concentrations of PM2.5 for longer periods of time than other nearby metropolitan areas. According to the EPA, Reno experienced 43 days of elevated PM2.5 (above 12 µg/m^3^) since 1 Aug 2020, while the San Francisco Bay Area in California experienced only 26 days of elevated PM2.5.

Current recommendations from the centers for disease control and prevention (CDC) with regards to wildfire smoke during the pandemic recognize the possibility that wildfire smoke may exacerbate the risk of SARS-CoV-2 infection. However, the EPA does not currently provide specific recommendations for wildfire smoke protection during the pandemic. Overall, the recommendations are to stay inside, use air cleaners, and wear N95 (or P100) respirators. Some of these recommendations may not be helpful during a pandemic that requires social distancing and limits the availability of personal protective equipment (ref. [21]).

Even with the possibility of COVID-19 vaccines becoming widely available during the first half of 2021, this year’s wildfire season may co-occur with an ongoing pandemic. Thus, our findings should help shape regional policies that seek to manage the combined threats of wildfires and the pandemic. These policies might include lowering the recommended healthy limit for PM2.5 in cities with a high prevalence of SARS-CoV-2, establishing “clean air” shelters that maintain social distancing, and allocating sufficient quantities of appropriate respirators to areas at high risk for wildfires.

## Supplementary information

Supplementary material
