# Supplementary material for: SARS-CoV-2 test positivity rate in Reno, Nevada: association with PM2.5 during the 2020 wildfire smoke events in the western United States
Source: J Expo Sci Environ Epidemiol. 2021 Jul 13;31(5):797–803. doi: 10.1038/s41370-021-00366-w (PMC8276229; doi:10.1038/s41370-021-00366-w)
Supplement: Supplementary file 1 — Supplementary material [file 41370_2021_366_MOESM1_ESM.docx]

**Supplementary material**

**SARS-CoV-2 test positivity rate in Reno, Nevada: association with PM2.5 during the 2020 wildfire smoke events in the western United States**

Daniel Kiser, Gai Elhanan, William J. Metcalf, Brendan Schnieder, Joseph J. Grzymski

| **Fire Name** | **Latitude** | **Longitude** | **Start**  **Date** | **End**  **Date** | **Acres** | **Source** |
| --- | --- | --- | --- | --- | --- | --- |
| August Complex | 39.625 | -122.809 | 17-Aug | 15-Nov | 1,032,648 | https://inciweb.nwcg.gov/incident/6983/ |
| SCU Lightning Complex | 37.439 | -121.304 | 16-Aug | 1-Oct | 396,624 | https://www.fire.ca.gov/incidents/2020/8/18/scu-lightning-complex/ |
| Creek Fire | 37.201 | -119.272 | 4-Sep | 31-Dec | 379,895 | https://inciweb.nwcg.gov/incident/7147 |
| LNU Lightning Complex | 38.549 | -122.506 | 17-Aug | 2-Oct | 363,220 | https://inciweb.nwcg.gov/incident/7027/ |
| North Complex | 40.091 | -120.391 | 17-Aug | 30-Nov | 318,935 | https://inciweb.nwcg.gov/incident/6997/ |
| SQF Complex | 36.255 | -118.497 | 19-Aug | 5-Jan | 174,178 | https://inciweb.nwcg.gov/incident/7048/ |
| Slater/Devil Fires | 41.766 | -123.375 | 8-Oct | 15-Nov | 157,229 | https://inciweb.nwcg.gov/incident/7173/ |
| Red Salmon Complex | 41.185 | -123.433 | 27-Jul | 23-Nov | 144,698 | https://inciweb.nwcg.gov/incident/6891/ |
| CZU Lightning Fire | 37.262 | -122.223 | 16-Aug | 22-Sep | 86,509 | https://www.fire.ca.gov/incidents/2020/8/16/czu-lightning-complex-including-warnella-fire/ |
| W-5 Cold Springs Fire | 41.029 | -120.281 | 18-Aug | 15-Sep | 84,817 | https://inciweb.nwcg.gov/incident/7010/ |
| July Complex | 41.699 | -121.477 | 22-Jul | 5-Aug | 83,261 | https://inciweb.nwcg.gov/incident/article/6881/52815/ |
| Zogg Fire | 40.539 | -122.566 | 27-Sep | 13-Oct | 56,338 | https://www.fire.ca.gov/incidents/2020/9/27/zogg-fire/ |
| Loyalton Fire | 39.681 | -120.171 | 14-Aug | 14-Sep | 47,029 | https://inciweb.nwcg.gov/incident/6975/ |
| Slink Fire | 38.568 | -119.568 | 29-Aug | 22-Oct | 26,759 | https://inciweb.nwcg.gov/incident/7105/ |
| Gold Fire | 41.113 | -120.921 | 20-Jul | 12-Aug | 22,634 | https://www.fire.ca.gov/incidents/2020/7/20/gold-fire/ |
| Numbers Fire | 38.843 | -119.639 | 6-Jul | 14-Jul | 18,380 | https://inciweb.nwcg.gov/incident/6833/ |
| Poodle Fire | 40.818 | -119.648 | 12-Aug | 19-Aug | 13,606 | https://inciweb.nwcg.gov/incident/6954/ |
| Baccarat Fire | 39.784 | -119.952 | 4-Oct | 8-Oct | 10,520 | https://twitter.com/TMFPD/status/1313912210498830336 |
| Poeville Fire | 39.576 | -119.913 | 26-Jun | 2-Jul | 2,975 | https://inciweb.nwcg.gov/incident/6810/ |
| Fork Fire | 38.990 | -120.394 | 8-Sep | 9-Nov | 1,673 | https://inciweb.nwcg.gov/incident/7165/ |
| Hog Fire | 36.875 | -119.302 | 12-Jun | 18-Jun | 533 | https://inciweb.nwcg.gov/incident/6765/ |

Table S1 – Locations (latitude/longitude) and dates of major wildfires occurring in the region during our study period, 15 May to 20 Oct 2020, that likely impacted air quality in Reno, Nevada.

Notes: Latitudes and longitudes are best estimates of the midpoints or ignition points. Complex fires may have a midpoint in an area that did not burn. End dates indicate when the fire was contained, but the fire may have continued to burn or smolder for a longer period. All fires are marked in figure 1 except the SQF Complex, which was furthest south. The North Complex Fire was responsible for the highest air pollution episodes in Reno.

Table S2 **–** Percent change in the SARS-CoV-2 test positivity rate associated with a 10 𝜇g/m^3^ increase in PM2.5.

|  |  |  | **Averages of daily PM2.5** | |  | **Distributed lag models** | |
| --- | --- | --- | --- | --- | --- | --- | --- |
| **Lag** | **Single-day PM2.5** |  | **Three-day** | **Seven-day** |  | **Quadratic** | **Cubic** |
| 0 | 1.2% (-1.5, 3.9) |  | 3.1% (0.0, 6.3)* | 6.3% (2.5, 10.3)* |  | -0.1% (-2.1, 2.0) | -0.1% (-2.2, 2.1) |
| 1 | 2.1% (-0.5, 4.8) |  | NA | NA |  | 0.5% (-0.5, 1.6) | 0.6% (-0.4, 1.7) |
| 2 | 3.3% (0.7, 5.9)* |  | NA | NA |  | 0.9% (0.3, 1.6)* | 1.0% (0.2, 1.9)* |
| 3 | 3.4% (0.8, 6.1)* |  | 5.0% (2.0, 8.1)* | NA |  | 1.2% (0.3, 2.0)* | 1.2% (0.3, 2.1)* |
| 4 | 3.7% (1.2, 6.4)* |  | NA | NA |  | 1.2% (0.3, 2.2)* | 1.2% (0.3, 2.1)* |
| 5 | 3.3% (0.7, 5.9)* |  | NA | NA |  | 1.1% (0.3, 2.0)* | 1.0% (0.3, 1.7)* |
| 6 | 2.5% (0.0, 5.1)* |  | 2.3% (-0.8, 5.4) | NA |  | 0.9% (0.2, 1.5)* | 0.8% (0.2, 1.4)* |
| 7 | 2.1% (-0.5, 4.8) |  | NA | -1.2% (-4.7, 2.5) |  | 0.4% (-0.6, 1.4) | 0.5% (-0.2, 1.2) |
| 8 | 0.2% (-2.4, 2.8) |  | NA | NA |  | -0.2% (-2.2, 1.8) | 0.2% (-0.7, 1.0) |
| 9 | -0.4% (-2.8, 2.2) |  | -1.1% (-3.9, 1.8) | NA |  | NA | 0.0% (-0.9, 0.9) |
| 10 | -0.8% (-3.2, 1.7) |  | NA | NA |  | NA | -0.2% (-1.0, 0.7) |
| 11 | -1.6% (-4.0, 0.9) |  | NA | NA |  | NA | -0.1% (-1.1, 0.9) |
| 12 | -1.9% (-4.3, 0.5) |  | -3.2% (-6.0, -0.4)* | NA |  | NA | 0.1% (-1.9, 2.1) |
| 13 | -2.7% (-5.0, -0.3)* |  | NA | NA |  | NA | NA |
| 14 | -2.7% (-5.0, -0.3)* |  | NA | -1.2% (-4.6, 2.3) |  | NA | NA |

*Statistically significant (p < 0.05)

Note: Percents indicate relative changes and not absolute changes in the positivity rate. These estimates are plotted in Figure 2 (for single-day PM2.5, as well as three- and seven-day averages of PM2.5) and Figure 3 (for quadratic and cubic distributed lag models).

Table S3 – Results of sensitivity analysis 1: Percent change in the SARS-CoV-2 test positivity rate associated with a 10 𝜇g/m^3^ increase in PM2.5, when we increased the maximum degrees of freedom from four to nine for the smooth of time in the base model.

|  |  |  | **Averages of daily PM2.5** | |  | **Distributed lag models** | |
| --- | --- | --- | --- | --- | --- | --- | --- |
| **Lag** | **Single-day PM2.5** |  | **Three-day** | **Seven-day** |  | **Quadratic** | **Cubic** |
| 0 | 1.1% (-1.5, 3.8) |  | 3.1% (0.0, 6.3) | 6.4% (2.5, 10.4)* |  | -0.1% (-2.1, 2.0) | -0.2% (-2.3, 2.0) |
| 1 | 2.1% (-0.5, 4.8) |  | NA | NA |  | 0.5% (-0.5, 1.6) | 0.6% (-0.5, 1.7) |
| 2 | 3.2% (0.7, 5.9)* |  | NA | NA |  | 1.0% (0.3, 1.6)* | 1.0% (0.1, 1.8)* |
| 3 | 3.4% (0.8, 6.1)* |  | 4.9% (1.9, 8.1)* | NA |  | 1.2% (0.4, 2.0)* | 1.2% (0.3, 2.1)* |
| 4 | 3.7% (1.1, 6.3)* |  | NA | NA |  | 1.3% (0.3, 2.2)* | 1.2% (0.3, 2.0)* |
| 5 | 3.1% (0.6, 5.7)* |  | NA | NA |  | 1.1% (0.3, 2.0)* | 1.0% (0.3, 1.7)* |
| 6 | 2.4% (-0.1, 5.0) |  | 2.0% (-1.0, 5.2) | NA |  | 0.8% (0.2, 1.5)* | 0.8% (0.2, 1.4)* |
| 7 | 2.0% (-0.6, 4.6) |  | NA | -1.9% (-5.4, 1.9) |  | 0.4% (-0.6, 1.4) | 0.5% (-0.2, 1.1) |
| 8 | 0.0% (-2.6, 2.6) |  | NA | NA |  | -0.3% (-2.3, 1.7) | 0.2% (-0.7, 1.0) |
| 9 | -0.5% (-3.0, 2.0) |  | -1.3% (-4.2, 1.6) | NA |  | NA | -0.1% (-1.0, 0.8) |
| 10 | -0.9% (-3.4, 1.6) |  | NA | NA |  | NA | -0.2% (-1.1, 0.6) |
| 11 | -1.8% (-4.2, 0.7) |  | NA | NA |  | NA | -0.3% (-1.3, 0.8) |
| 12 | -2.1% (-4.5, 0.4) |  | -3.8% (-6.6, -1.0)* | NA |  | NA | -0.1% (-2.0, 2.0) |
| 13 | -2.9% (-5.3, -0.6)* |  | NA | NA |  | NA | NA |
| 14 | -3.1% (-5.4, -0.8)* |  | NA | -2.2% (-5.8, 1.4) |  | NA | NA |

*Statistically significant (p < 0.05)

Note: Intended for comparison with Table S1. Percents indicate relative changes and not absolute changes in the positivity rate.

Table S4 – Results of sensitivity analysis 2: Percent change in the SARS-CoV-2 test positivity rate associated with a 10 𝜇g/m^3^ increase in PM2.5, when we added the seven-day average of relative humidity as a predictor in the base model.

|  |  |  | **Averages of daily PM2.5** | |  | **Distributed lag models** | |
| --- | --- | --- | --- | --- | --- | --- | --- |
| **Lag** | **Single-day PM2.5** |  | **Three-day** | **Seven-day** |  | **Quadratic** | **Cubic** |
| 0 | 1.2% (-1.5, 3.9) |  | 3.2% (0.1, 6.4)* | 6.4% (2.5, 10.3)* |  | -0.1% (-2.1, 2.1) | -0.1% (-2.2, 2.1) |
| 1 | 2.2% (-0.5, 4.9) |  | NA | NA |  | 0.5% (-0.5, 1.6) | 0.6% (-0.4, 1.7) |
| 2 | 3.3% (0.7, 6.0)* |  | NA | NA |  | 0.9% (0.3, 1.6)* | 1.0% (0.2, 1.9)* |
| 3 | 3.4% (0.8, 6.1)* |  | 5.0% (2.0, 8.2)* | NA |  | 1.2% (0.3, 2.0)* | 1.2% (0.3, 2.1)* |
| 4 | 3.7% (1.1, 6.4)* |  | NA | NA |  | 1.2% (0.3, 2.2)* | 1.2% (0.3, 2.1)* |
| 5 | 3.3% (0.7, 5.9)* |  | NA | NA |  | 1.1% (0.3, 2.0)* | 1.0% (0.3, 1.7)* |
| 6 | 2.5% (0.0, 5.2) |  | 2.3% (-0.8, 5.5) | NA |  | 0.9% (0.2, 1.5)* | 0.8% (0.1, 1.4)* |
| 7 | 2.1% (-0.5, 4.8) |  | NA | -1.3% (-4.9, 2.5) |  | 0.4% (-0.6, 1.4) | 0.5% (-0.2, 1.2) |
| 8 | 0.2% (-2.5, 2.9) |  | NA | NA |  | -0.2% (-2.2, 1.8) | 0.2% (-0.7, 1.0) |
| 9 | -0.5% (-3.0, 2.1) |  | -1.3% (-4.2, 1.7) | NA |  | NA | -0.1% (-1.0, 0.9) |
| 10 | -1.0% (-3.5, 1.6) |  | NA | NA |  | NA | -0.2% (-1.0, 0.7) |
| 11 | -1.9% (-4.3, 0.7) |  | NA | NA |  | NA | -0.1% (-1.2, 0.9) |
| 12 | -2.0% (-4.5, 0.4) |  | -3.3% (-6.1, -0.5)* | NA |  | NA | 0.1% (-1.9, 2.1) |
| 13 | -2.7% (-5.1, -0.3)* |  | NA | NA |  | NA | NA |
| 14 | -2.7% (-5.1, -0.3)* |  | NA | -1.3% (-4.8, 2.4) |  | NA | NA |

*Statistically significant (p < 0.05)

Note: Intended for comparison with Table S1. Percents indicate relative changes and not absolute changes in the positivity rate.

Table S5 – Results of sensitivity analysis 3: Percent change in the SARS-CoV-2 test positivity rate associated with a 10 𝜇g/m^3^ increase in PM2.5, when we replaced the seven-day average of temperature in the base model with the three-day average of temperature.

|  |  |  | **Averages of daily PM2.5** | |  | **Distributed lag models** | |
| --- | --- | --- | --- | --- | --- | --- | --- |
| **Lag** | **Single-day PM2.5** |  | **Three-day** | **Seven-day** |  | **Quadratic** | **Cubic** |
| 0 | 1.1% (-1.6, 3.8) |  | 2.9% (-0.1, 6.1) | 6.0% (2.2, 10.0)* |  | -0.2% (-2.3, 1.9) | -0.2% (-2.3, 1.9) |
| 1 | 2.0% (-0.6, 4.7) |  | NA | NA |  | 0.4% (-0.6, 1.5) | 0.5% (-0.5, 1.6) |
| 2 | 3.1% (0.5, 5.7)* |  | NA | NA |  | 0.9% (0.3, 1.5)* | 1.0% (0.1, 1.8)* |
| 3 | 3.2% (0.6, 5.9)* |  | 4.8% (1.8, 8.0)* | NA |  | 1.2% (0.3, 2.0)* | 1.1% (0.2, 2.1)* |
| 4 | 3.6% (1.0, 6.3)* |  | NA | NA |  | 1.3% (0.3, 2.2)* | 1.1% (0.2, 2.0)* |
| 5 | 3.2% (0.6, 5.8)* |  | NA | NA |  | 1.1% (0.3, 2.0)* | 1.0% (0.2, 1.7)* |
| 6 | 2.4% (-0.2, 5.0) |  | 2.0% (-1.1, 5.1) | NA |  | 0.8% (0.1, 1.5)* | 0.7% (0.1, 1.3)* |
| 7 | 1.9% (-0.7, 4.6) |  | NA | -1.4% (-4.9, 2.2) |  | 0.3% (-0.7, 1.3) | 0.4% (-0.3, 1.1) |
| 8 | 0.0% (-2.6, 2.6) |  | NA | NA |  | -0.4% (-2.4, 1.6) | 0.1% (-0.7, 1.0) |
| 9 | -0.5% (-3.0, 2.0) |  | -1.2% (-4.1, 1.7) | NA |  | NA | -0.1% (-1.0, 0.8) |
| 10 | -0.9% (-3.3, 1.6) |  | NA | NA |  | NA | -0.3% (-1.1, 0.6) |
| 11 | -1.7% (-4.1, 0.8) |  | NA | NA |  | NA | -0.2% (-1.2, 0.8) |
| 12 | -1.9% (-4.3, 0.5) |  | -3.3% (-6.0, -0.4)* | NA |  | NA | 0.0% (-2.0, 2.0) |
| 13 | -2.7% (-5.0, -0.3)* |  | NA | NA |  | NA | NA |
| 14 | -2.7% (-5.0, -0.3)* |  | NA | -1.1% (-4.6, 2.4) |  | NA | NA |

*Statistically significant (p < 0.05)

Note: Intended for comparison with Table S1. Percents indicate relative changes and not absolute changes in the positivity rate.

Table S6 – Excess COVID-19 cases due to wildfire smoke estimated by the main analysis versus the sensitivity analyses.

|  | **Excess COVID-19 cases** | |
| --- | --- | --- |
|  | **Count (95% CI)** | **Percent (95% CI)** |
| Main analysis | 178 (149, 198) | 17.7% (14.4, 20.1) |
| **Sensitivity analyses** |  |  |
| Analysis 1 | 172 (141, 193) | 17.0% (13.6, 19.5) |
| Analysis 2 | 179 (148, 199) | 17.9% (14.3, 20.3) |
| Analysis 3 | 175 (146, 195) | 17.4% (14.1, 19.8) |

CI: confidence interval

Note: The time period during which excess cases were estimated was 16 Aug to 10 Oct 2020 for all analyses. Sensitivity analysis 1 increased maximum degrees of freedom for the smooth of time from 4 to 9. Sensitivity analysis 2 included the seven-day average of humidity, in addition to the predictors used in the main analysis. Sensitivity analysis 3 replaced the seven-day average of temperature with a three-day average of temperature.


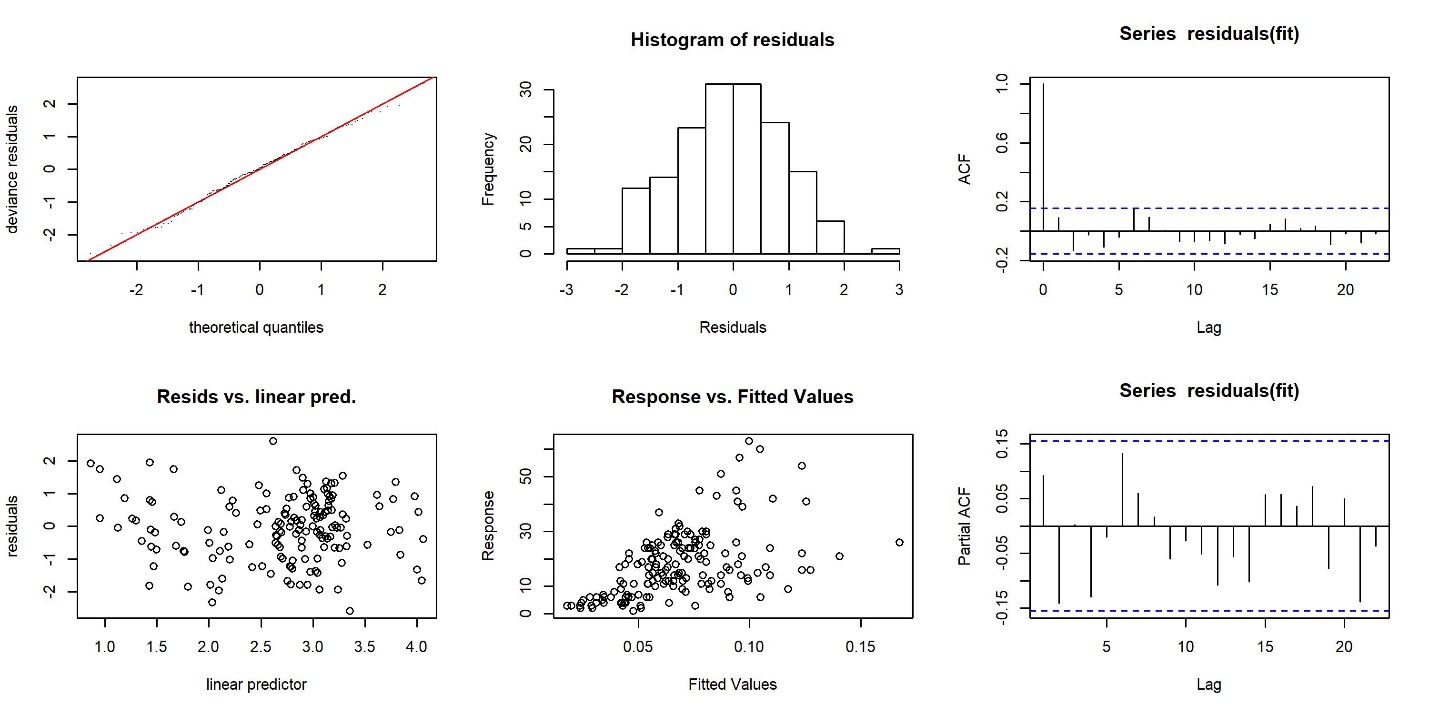


Figure S1 – Diagnostic plots for the model including the seven-day rolling average of PM2.5, as produced by the R package **mgcv** (Wood 2017) function *gam.check()* and the R base functions *acf()* and *pacf()*. Clockwise from upper left: QQ-plot, histogram of residuals, autocorrelation function plot, residuals versus linear predictors, response versus fitted values, and partial autocorrelation function plot. These plots indicate that the distribution of the residuals reasonably follows a normal distribution, there is no heteroscedasticity, and the residuals are not significantly autocorrelated. Diagnostic plots for other models were similar.


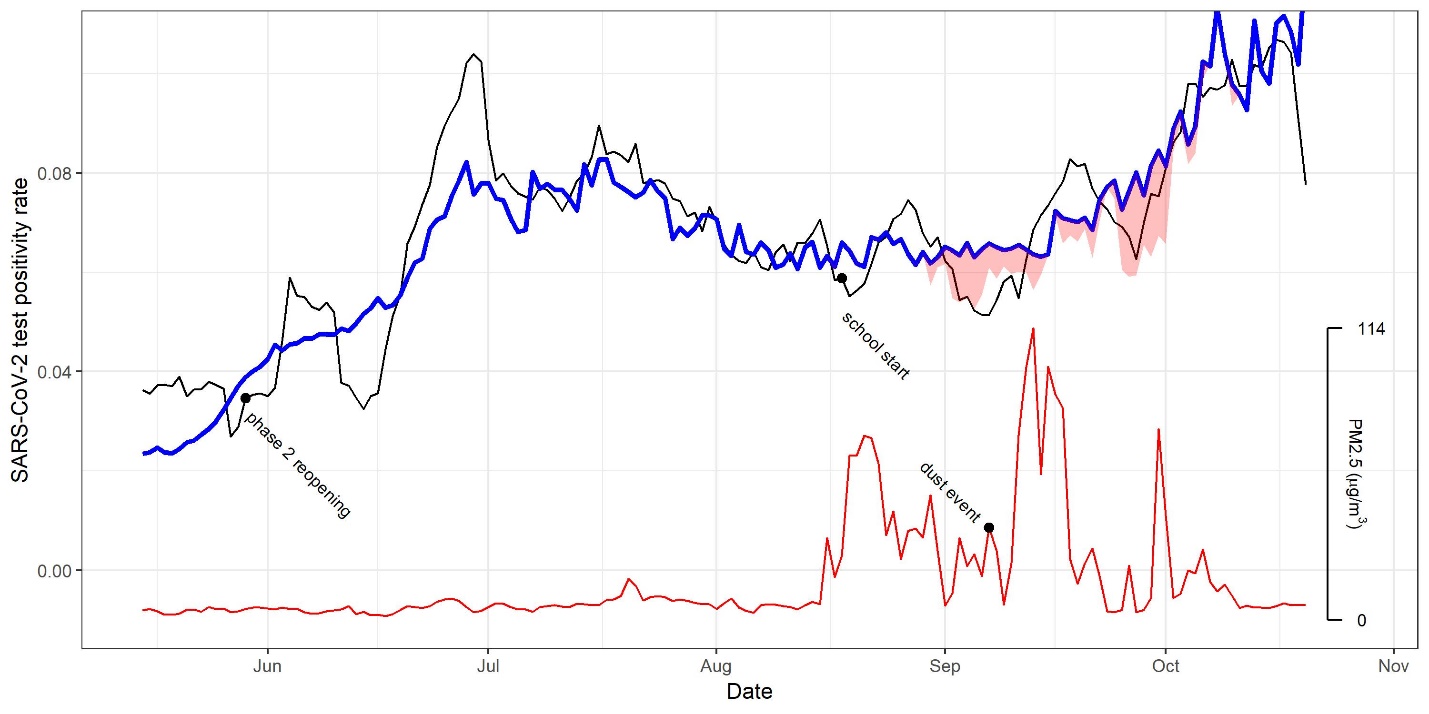


Figure S2 – SARS-CoV-2 test positivity rate from 15 May to 20 Oct 2020. Expected positivity rates were estimated by the model using lag 14 of the single-day mean PM2.5 as a predictor. The black line indicates the seven-day moving average of the positivity rate (each day averaged with the three days prior and the three days following). The blue line indicates the expected positivity rate if the concentration of lag-14 PM2.5 during the period 16 Aug to 10 Oct 2020 had remained at the average level for the same time period in 2019 (4.8 μg/m^3^). The red-shaded region indicates the expected positivity rate based on the actual PM2.5, which is indicated by the red line. For this figure, weekday effects were removed from the model estimates for clarity.

**References**

Wood S. *Generalized Additive Models: An Introduction with R*. 2nd ed. Boca Raton: Chapman & Hall/CRC; 2017.
